# Association of FUT2 rs601338 Genotype with Colonic Mucosal Microbiome Composition, Post-Transplant Bacteremia, and All-Cause Mortality After Liver Transplantation for Primary Sclerosing Cholangitis: A Retrospective Cohort Study †

**DOI:** 10.3390/jcm15124755

**Published:** 2026-06-18

**Authors:** Ruslan A. Mammadov, Henk P. Roest, Gwenny M. Fuhler, Junhong Su, Thijmen Visseren, Harry L. A. Janssen, Robert J. Porte, Sarwa Darwish Murad, Bettina E. Hansen, Luc J. W. van der Laan, Maikel P. Peppelenbosch

**Affiliations:** 1 Department of Surgery, Erasmus MC Transplant Institute, Erasmus MC—University Medical Center, 3015 GD Rotterdam, The Netherlands; r.mammadov@erasmusmc.nl (R.A.M.); h.roest@erasmusmc.nl (H.P.R.); r.j.porte@erasmusmc.nl (R.J.P.); 2 Department of Gastroenterology and Hepatology, Erasmus MC—Erasmus University Medical Center, 3015 GD Rotterdam, The Netherlands; g.fuhler@erasmusmc.nl (G.M.F.); j.su.1@erasmusmc.nl (J.S.); t.visseren@erasmusmc.nl (T.V.); h.janssen@erasmusmc.nl (H.L.A.J.); s.darwishmurad@erasmusmc.nl (S.D.M.); 3 Department of Epidemiology, Erasmus MC—Erasmus University Medical Center, 3015 GD Rotterdam, The Netherlands; b.hansen@erasmusmc.nl

**Keywords:** primary sclerosing cholangitis, fucosyltransferase-2, FUT2 polymorphism, gut microbiome, bacteremia, liver transplantation, mucosal microbiota

## Abstract

**Background/Objectives:** Primary sclerosing cholangitis (PSC) is a chronic cholestatic liver disease frequently requiring liver transplantation (LTx). The gut–liver axis, host genetics, and microbial dysbiosis are thought to contribute to disease progression and post-transplant outcomes. The FUT2 rs601338 polymorphism influences mucosal fucosylation, host–microbial interactions, and susceptibility to infection. This study aimed to investigate the association between FUT2 genotype, colonic mucosal microbiome composition, post-transplant bacteremia, and all-cause mortality in a retrospective single-center PSC cohort. **Methods:** This retrospective cohort study included PSC patients who underwent LTx at Erasmus MC University Medical Center (Rotterdam, The Netherlands) between 1987 and 2015. Pre-transplant archival formalin-fixed paraffin-embedded (FFPE) colonic biopsy specimens were available for microbiome analysis. Of 169 transplanted patients, FFPE tissue was available for 98 individuals, and FUT2 rs601338 genotyping was successfully performed in 87 patients. Patients were classified as FUT2 non-secretors (AA, *n* = 28) and secretors (GA/GG, *n* = 59). Post-transplant bacteremia was assessed based on clinically indicated blood cultures during follow-up. Colonic mucosal microbiome composition was analyzed using 16S rRNA gene sequencing. **Results:** FUT2 non-secretors showed a distinct colonic mucosal microbiome profile compared with secretors, characterized by differential abundance of selected taxa within Proteobacteria, Firmicutes, and Bacteroidetes. Post-transplant bacteremia occurred in 30 patients and was more frequent among non-secretors (43%) compared with secretors (15%). Both FUT2 non-secretor status and post-transplant bacteremia were associated with reduced all-cause post-transplant survival in Kaplan–Meier analysis and remained associated with mortality in multivariable regression models. Specific microbial taxa were also showed associations with bacteremia, mortality, and established prognostic scores, including the Amsterdam–Oxford Model and Mayo Risk Score. **Conclusions:** FUT2 genotype is associated with alterations in colonic mucosal microbiome composition, post-transplant bacteremia, and all-cause mortality in PSC patients undergoing liver transplantation. These findings suggest a potential interplay between host genetics, intestinal microbiota, and infectious complications after transplantation. Given the retrospective design, limited sample size, and use of archival FFPE tissue, all findings should be interpreted as exploratory and hypothesis-generating. Prospective multicenter studies using standardized sampling and high-resolution metagenomic approaches are warranted for validation.

## 1. Introduction

### Revised Introduction (with AOM Integration)

Primary sclerosing cholangitis (PSC) is a chronic, progressive cholestatic liver disease characterized by inflammation and fibrosis of the intra- and extrahepatic bile ducts, ultimately leading to cirrhosis and liver failure. The only curative treatment is still liver transplantation (LTx), although post-transplant complications, including infectious events and disease recurrence, remain clinically relevant challenges [1,2,3].

PSC is strongly associated with inflammatory bowel disease (IBD), particularly ulcerative colitis, with reported co-occurrence in approximately 70% of patients, although prevalence varies depending on geographic region and diagnostic ascertainment [4,5,6]. While PSC-associated dysbiosis and IBD-associated dysbiosis overlap, they represent partially distinct microbial and immunological phenotypes. IBD is typically characterized by inflammation-driven microbial shifts secondary to mucosal immune activation, whereas PSC is increasingly recognized as a disorder of the gut–liver axis in which intestinal barrier dysfunction, microbial translocation, and hepatobiliary immune responses play central roles in disease progression [6,7,8].

Recent work continues to strengthen the concept that PSC is tightly linked to persistent gut–liver axis dysregulation and mucosa-associated microbial alterations that may persist even after liver transplantation [9,10].

The gut–liver axis describes the bidirectional interaction between the intestinal microbiome, gut barrier integrity, and hepatic immune and metabolic function via the portal circulation. Disruption of this axis has been implicated in chronic liver diseases, including PSC, through mechanisms involving bacterial translocation, endotoxemia, and immune activation in genetically predisposed individuals. Recent studies have further highlighted the importance of host–microbiome interactions in shaping disease severity, transplant outcomes, and infection susceptibility [11,12,13].

A key host genetic factor implicated in PSC is the FUT2 gene, which encodes fucosyltransferase 2, an enzyme responsible for the secretion of fucosylated glycans on mucosal epithelial surfaces. These glycans play an important role in shaping microbial colonization, maintaining epithelial barrier integrity, and modulating host–microbial interactions. The common European variant rs601338 (G > A) leads to a non-secretor phenotype in homozygous carriers (AA), resulting in absent or markedly reduced mucosal fucosylation. Importantly, this variant does not capture all functional FUT2 variation across non-European populations but represents the predominant loss-of-function allele in European ancestry cohorts [14,15].

Although recent reviews on host–microbiome interactions in transplantation emphasize the importance of genetic determinants such as FUT2 in shaping microbial colonization patterns and immune responses, direct evidence linking *FUT2* genotype to mucosal microbiome composition in PSC transplant cohorts remains limited [16].

Previous studies have linked *FUT2* non-secretor status to altered gut microbiome composition, increased susceptibility to infections, and reduced transplant-free survival in PSC. However, these studies have primarily focused on fecal microbiome profiling and have not integrated mucosal microbiome data or post-transplant infectious outcomes in a liver transplantation setting. Furthermore, the mechanistic relationship between *FUT2* genotype, microbial composition at the colonic mucosal surface, and systemic infectious complications such as bacteremia remains insufficiently understood [8,17,18,19].

The gut microbiota plays a central role in regulating immune homeostasis, epithelial barrier integrity, and resistance to pathogen colonization. Disruption of these functions may facilitate bacterial translocation and lead to bacteremia, which is a clinically relevant complication after liver transplantation and a major contributor to morbidity and mortality. However, it remains unclear whether host *FUT2* genotype is associated with specific mucosal microbial signatures that predispose to post-transplant bacteremia and adverse clinical outcomes [20,21,22].

Importantly, no previous studies have simultaneously integrated (i) colonic mucosal microbiome profiling, (ii) *FUT2* rs601338 genotyping, (iii) post-transplant bacteremia, and (iv) long-term survival outcomes in PSC patients undergoing liver transplantation. Additionally, the relationship between microbial signatures and established prognostic models such as the Amsterdam–Oxford Model (AOM) and the Mayo Risk Score has not been explored in this context. The Amsterdam–Oxford Model (AOM) is a validated prognostic model for PSC developed using Dutch and UK population cohorts and incorporates age, albumin, platelet count, total bilirubin, alkaline phosphatase, aspartate aminotransferase, and PSC subtype characteristics. Compared with the Mayo Risk Score (MRS), the AOM was designed to improve long-term prognostic stratification across broader PSC populations, although both models have recognized limitations regarding individualized outcome prediction and external validation in heterogeneous patient cohorts [23,24,25].

Based on these gaps, we hypothesized that *FUT2* genotype is associated with distinct colonic mucosal microbiome profiles in PSC patients prior to liver transplantation, and that these microbial patterns are linked to post-transplant bacteremia and all-cause mortality independently of recurrent PSC. We further explored whether microbiome features correlate with established clinical prognostic models, aiming to identify potential microbiome-associated biomarkers of disease severity and post-transplant outcome [24,25,26].

## 2. Materials and Methods

### 2.1. Patients

This retrospective single-center cohort study included patients with Primary Sclerosing Cholangitis (PSC) who underwent liver transplantation (LTx) at the Erasmus MC University Medical Center Rotterdam, The Netherlands, between 1987 and 2015 [24].

This cohort has been partially used in prior studies focusing on PSC recurrence and microbiome characterization; however, the present analysis represents a novel integration of FUT2 genotyping, mucosal microbiome profiling, post-transplant bacteremia, and long-term survival outcomes, which have not been previously reported.

The study was conducted using pre-transplant archival formalin-fixed paraffin-embedded (FFPE) colonic biopsy specimens obtained during routine surveillance colonoscopy.

#### 2.1.1. Inclusion and Exclusion Criteria

Eligible patients were adults (≥18 years) diagnosed with primary sclerosing cholangitis (PSC) who underwent liver transplantation (LTx) at the Erasmus MC University Medical Center Rotterdam between 1987 and 2015. Only patients undergoing first-time liver transplantation were included; re-transplantation cases were excluded to avoid confounding related to graft failure and repeated immunosuppression exposure. Inclusion required availability of pre-transplant colonic mucosal formalin-fixed paraffin-embedded (FFPE) biopsy material obtained during routine surveillance colonoscopy. Patients with a history of total colectomy or subtotal colectomy resulting in insufficient colonic tissue for microbiome analysis were excluded. Patients were included only if biopsies were obtained during clinically stable disease, defined as absence of active cholangitis or other acute infectious episodes at the time of sampling. In line with institutional practice, patients presenting with active cholangitis requiring antibiotic treatment were not scheduled for elective transplantation and therefore did not undergo screening colonoscopy during such episodes.

Patients receiving systemic antibiotic therapy within 4 weeks prior to biopsy collection were excluded to minimize antibiotic-related microbiome perturbation. Immunosuppressive therapy was not present at the time of biopsy, as all samples were obtained in the pre-transplant setting. Patients with active severe inflammatory bowel disease requiring hospitalization or escalation of immunosuppressive or antibiotic therapy at the time of biopsy were excluded; however, patients with clinically quiescent IBD were included. FFPE samples were excluded from downstream analyses if DNA extraction failed or yielded insufficient host genomic DNA for *FUT2* genotyping. Microbiome sequencing data were excluded if sequencing depth was below 1200 reads after quality control filtering, or if contamination thresholds (kitome-derived OTUs > 1%) were exceeded. After application of these criteria, 87 patients with complete *FUT2* genotyping and microbiome data were included in the final analysis.

Due to the retrospective nature of the study and the long-term biobank storage of formalin-fixed paraffin-embedded (FFPE) samples collected between 1987 and 2015, detailed information on fixation duration and exact storage time per individual block was not consistently available and therefore could not be included in the analysis.

In order to strengthen the reliability of our data and a more comprehensive assessment of long-term outcomes, extended follow-up times were included. All patients included in this study had previously undergone a screening colonoscopy with surveillance biopsies before LTx and were included based on the availability of banked pre-transplant FFPE biopsies obtained via colonoscopy. From all PSC patients, whether or not they had concurrent Inflammatory Bowel Disease (IBD), random samples were routinely collected from 10 different sites for pathological evaluation of underlying dysplasia. These biopsy materials were preserved under stable conditions as formalin-fixed paraffin-embedded (FFPE) blocks. Preference was given to samples from the right colon. The number of biopsies per patient was standardized to one representative FFPE biopsy per individual for microbiome and host DNA analyses, preferentially selected from the right colon to reduce inter-segmental microbiome heterogeneity. Biopsy selection was performed using a predefined hierarchy (right colon preferred), independently of the *FUT2* genotype, clinical outcome, and microbiome results. Additionally, we retrieved information on clinical outcomes and the presence of positive bacterial blood cultures from electronic patient files for all patients with mucosal colon biopsies. Blood cultures were obtained throughout the post-transplant follow-up period in accordance with the institution’s standard clinical practice. The timing and frequency of sampling were determined by the treating physicians based on clinical indications.

The cohort consisted of 87 PSC patients, reduced from the original 98-patient microbiome cohort described previously [23]. One patient was excluded due to low sequencing depth (<1200 reads), and 10 patients were excluded due to insufficient quantity or quality of host genomic DNA extracted from FFPE material, which precluded successful *FUT2* genotyping.

All patients provided written informed consent for the use of materials and clinical information for medical research, as approved by the Medical Ethical Committee of the Erasmus MC.

The Amsterdam–Oxford Model (AOM) and Mayo Risk Score were calculated using established published formulas [25]. Scores were derived from pre-transplant clinical and biochemical data obtained closest to the time of liver transplantation. Patients with incomplete data required for score calculation were excluded from the corresponding analyses (complete-case analysis).

#### 2.1.2. IBD Status, Disease Activity, and Mucosal Inflammation

Inflammatory bowel disease (IBD) status was defined based on established clinical and histopathological diagnosis recorded in the medical records. At the time of pre-transplant surveillance colonoscopy, patients were considered to be in a clinically quiescent state if they had no symptoms suggestive of active colitis and no clinical indication for escalation of IBD therapy, hospitalization, or treatment for acute flare. Colonoscopies were performed as part of routine surveillance rather than during episodes of clinically overt disease activity. However, systematic assessment of mucosal inflammation using standardized clinical activity indices (e.g., Mayo score), fecal calprotectin measurements, or uniformly applied endoscopic scoring systems was not available for the majority of patients due to the retrospective nature and extended inclusion period of the study. In addition, histological grading of inflammatory activity was not consistently reported in a structured and extractable format across all biopsy samples. Therefore, while overt active disease was unlikely at the time of sampling, subclinical or histological inflammation cannot be excluded and may have contributed to variability in mucosal microbiome composition.

### 2.2. Definition of Bacteremia

Bacteremia was defined as any positive blood culture determined by the clinical microbiology facility at the Erasmus Medical Center during the post-transplant follow-up period. Blood cultures were obtained in cases of fever (body temperature > 38.3 °C) or elevated infection parameters (such as C-reactive protein level and leukocyte count) as determined by responsible clinicians. In cases of suspected bacteremia, aerobic and anaerobic bottles were cultured for up to 5 days using the BD BACTEC FX Blood Culture System (BD Diagnostics, Breda, The Netherlands).

Positive blood cultures were inoculated onto Columbia blood agar with 5% sheep blood, MacConkey agar, chocolate agar, and Brucella blood agar (BD Diagnostics, Breda, The Netherlands). Bacterial isolates were further categorized into intestinal-origin bacteremia and non-intestinal-origin bacteremia. Bacterial species considered to be of intestinal origin included *Enterococcus faecalis*, *Enterococcus faecium*, *Bacteroides fragilis*, *Klebsiella pneumoniae*, *Citrobacter freundii*, *Enterobacter cloacae*, and *Escherichia coli*. Non-intestinal species included, among others, *Pseudomonas aeruginosa*, *Staphylococcus aureus*, *Staphylococcus epidermidis*, and *Streptococcus pyogenes*. This classification represents a pragmatic, organism-based proxy for gut-associated bacteremia rather than a definitive determination of infection source, as precise anatomical origin (e.g., biliary, urinary, catheter-related, or intra-abdominal) could not be reliably established in all cases within this retrospective cohort.

Blood cultures were obtained based on clinical indication during routine post-transplant care. Therefore, “no bacteremia” was defined as no documented positive blood culture in the clinical microbiology records during follow-up.

As blood cultures were not performed systematically in all patients, absence of bacteremia does not necessarily indicate absence of bloodstream infection, but rather the absence of documented or clinically suspected infection requiring microbiological testing. This introduces potential ascertainment bias in bacteremia classification.

The timing and frequency of blood culture sampling were determined by treating physicians based on clinical indication and were not standardized across patients.

### 2.3. Antibiotic and Immunosuppressive Exposure

All colonic biopsies used for microbiome profiling were obtained in the pre-transplant period during routine screening colonoscopy, performed when patients were in a clinically stable phase of PSC and IBD. According to the standard clinical practice at the Erasmus MC, systemic antibiotics are prescribed only on clinical indication (e.g., acute cholangitis) and are not given prophylactically prior to liver transplantation. Patients with signs of active cholangitis or active IBD requiring antibiotic therapy were not eligible for transplantation at that time and therefore did not undergo screening colonoscopy. At the time of colonic biopsy collection, all patients were in a clinically stable phase of disease and were not receiving systemic antibiotic therapy or immunosuppressive treatment for active infection or active inflammatory bowel disease. Patients requiring such treatments were not eligible for elective surveillance colonoscopy at that time and were therefore not included in the biopsy cohort.

Although detailed IBD activity scores (e.g., Mayo score) were not systematically available due to the retrospective nature of the cohort and long study period, biopsies were obtained exclusively during clinically quiescent disease states, minimizing confounding effects of active intestinal inflammation on microbiome composition.

### 2.4. Sample Collection, DNA Extraction, and 16S rRNA Gene Sequencing

We obtained patient DNA from FFPE blocks of biopsies obtained during colonoscopy. Microbial DNA analysis for this study was described previously [24], and repurposed and reanalyzed for the current study in relation to *FUT2* status and bacteremia, as well as changes in composition of the study population with the longer follow-up duration. Each FFPE specimen underwent slicing, with 14 slices of five microns taken for each biopsy, comprising seven superficial slices and seven mid-biopsy slices to cover most of the biopsy. Paraffin was removed using xylene. Subsequently, bacterial DNA was extracted from the colon biopsies using the RTP Bacteria DNA Mini Kit (Stratec^®^), Stratec Molecular GmbH, Berlin, Germany as per the manufacturer’s protocol [26].

For FFPE tissue processing, 5 µm sections were cut from paraffin blocks under sterile conditions. Paraffin was removed using two sequential xylene washes followed by graded ethanol washes (100%, 95%, and 70%) to ensure complete deparaffinization. Tissue pellets were then air-dried prior to enzymatic digestion. DNA extraction was performed using proteinase K-based lysis with incubation at 56 °C until complete tissue digestion, followed by heat inactivation at elevated temperature according to manufacturer’s instructions. DNA was subsequently purified using a column-based extraction kit (Stratec^®^ RTP Bacteria DNA Mini Kit). All samples were processed in batches using standardized protocols; however, detailed documentation of batch allocation, exact lysis duration per individual sample, and randomization across extraction runs was not consistently available due to the retrospective nature of the study.

DNA concentration and purity were assessed spectrophotometrically (NanoDrop™, Thermo Fisher Scientific, Waltham, MA, USA), revealing low-yield but amplifiable DNA, consistent with archival FFPE-derived samples.

An empty paraffin block not containing human tissue but undergoing the same DNA extraction protocol was used as a ‘kitome’ control to assess contamination.

Amplification of the bacterial 16S rRNA gene targeted the V3–V4 hypervariable regions using standard Illumina MiSeq-compatible primer sets as implemented by the sequencing provider (Macrogen^®^, Seoul, Republic of Korea). Sequencing libraries were prepared according to validated manufacturer-supported workflows for Illumina MiSeq paired-end sequencing. Detailed sequencing procedures, including platform-specific amplification chemistry and adapter incorporation, followed standardized protocols routinely used for 16S microbiome profiling and have been described previously [23]. Sequencing was performed on the Illumina MiSeq platform (Illumina Inc., San Diego, CA, USA; https://www.illumina.com) using paired-end reads targeting an expected amplicon size of approximately 460 bp.

Data were analyzed and displayed using QIIME V 1.9.1 (http://qiime.org) and Emperor (http://biocore.github.io/emperor accessed on 21 April 2026). Sequencing depth, rarefaction to 1200 reads, pipeline normalization, and multiple comparison corrections (FDR) were applied. The rarefaction threshold of 1200 reads was selected as a compromise to retain the maximum number of FFPE-derived samples while minimizing amplification noise inherent to low-biomass archival tissue.

OTUs representing more than 1% of total reads in the kitome control were removed. We observed a limited number of species in each patient group, which may impact the reliability of diversity analyses. These analyses generally require a minimal level of microbial diversity within each sample to produce robust results. Due to the low species count in some groups, diversity measures could have been skewed or were inaccurate, potentially leading to misleading conclusions. As a result, we refrained from performing alpha and beta diversity analyses to avoid overestimating or underestimating microbial diversity and to ensure the validity of our findings. To assess differences in microbiome composition between patient groups, Linear Discriminant Analysis (LDA) was employed to visualize variations at various taxonomic levels (phylum, class, order, family, and genus).

Bacterial taxa were identified through unbiased exploratory 16S rRNA sequencing analysis rather than preselected candidate organisms. Taxa discussed in detail were prioritized based on statistically significant differential abundance and their previously reported associations with PSC, IBD, intestinal barrier dysfunction, or systemic inflammation in prior literature and our earlier studies [8,24].

All microbiome findings derived from 16S rRNA sequencing were interpreted as exploratory and hypothesis-generating, given the known technical limitations of FFPE material, low microbial biomass, and the absence of shotgun metagenomic validation. Samples with sequencing depth below 1200 after quality filtering were excluded prior to downstream analysis.

### 2.5. FUT2 Genotyping

Genotyping for the rs601338 SNP on DNA extracted from FFPE blocks was carried out by LGC Genomics (Teddington, UK) using the PCR-based KASP (Kompetitive Allele Specific PCR) genotyping technology, with specific primers for the SNP. Host genomic DNA was extracted from FFPE sections using proteinase K digestion and column-based purification optimized for fragmented DNA, yielding sufficient DNA for PCR-based genotyping in 87 of 97 cases.

In cases where KASP genotyping results were inconclusive, the correct genotype was determined using PCR amplification and restriction fragment analysis. Specifically, KASP genotyping yielded definitive results in the majority of cases; a subset of samples with ambiguous or failed KASP calls underwent secondary RFLP analysis, resulting in successful genotype assignment in all remaining evaluable samples. The overall genotype call rate for the KASP assay was >95%, with well-separated fluorescence clusters enabling reliable genotype discrimination. RFLP analysis was used solely as a confirmatory method for samples with inconclusive KASP results and followed standard SNP validation workflows previously described in the literature for rs601338 genotyping.

Recipients with a homozygous mutation (AA) were classified as *FUT2* non-secretors, while recipients with GA or GG genotypes were considered *FUT2* secretors [8].

The observed rs601338 allele frequencies (A = 49.4%, G = 50.6%) were similar to those reported in European populations (~45–50% for the A allele).

### 2.6. Statistical Analysis

Statistical analysis was conducted using SPSS Statistics 25 (SPSS Inc., Chicago, IL, USA). Patient characteristics were described using means and standard deviations for normally distributed continuous variables, medians and ranges for non-normally distributed variables, and counts and proportions for categorical variables.

Analyses were performed for grouped *FUT2* genotypes (AA vs. GA + GG). For continuous data, comparisons were made using non-parametric Mann–Whitney U tests, and for categorical data, two-sided chi-square tests were applied. Hardy–Weinberg equilibrium for rs601338 was assessed using a chi-square test.

For the univariable analysis, binary logistic regression was applied to estimate crude odds ratios (ORs) with 95% confidence intervals (CIs). Variables with a *p*-value < 0.05 were considered statistically significant.

For the multivariable analysis, a binary logistic regression model was built to adjust for potential confounders. Variables with a *p*-value < 0.05 in the univariable analysis were included in the initial multivariable model. The Hosmer–Lemeshow test was used to evaluate the adequacy of the final model. Adjusted ORs with 95% CIs were reported, and statistical significance was set at *p* < 0.05.

Kaplan–Meier survival curves were generated using R software (version 4.2.2, R Project for Statistical Computing) with the survival and survminer packages to visualize differences in overall survival between groups. No time-to-event regression modelling was performed. Overall survival was analyzed as a binary outcome (death during follow-up: yes/no), rather than as a time-to-event endpoint, due to heterogeneity in follow-up duration across the cohort.

To evaluate the potential impact of the long study inclusion period on cohort characteristics and microbiome analyses, exploratory sensitivity analyses were performed using transplant year as a surrogate marker of transplant era. Associations between transplant year and selected clinical variables, including gender, IBD status, IBD medication, transplantation indication, rPSC, PSC type, donor type and biliary anastomosis were assessed using appropriate univariate statistical tests. To evaluate potential temporal effects on microbial community composition, principal component analysis (PCA) and Bray–Curtis-based principal coordinates analysis (PCoA) were performed. Differences in PC1 and PC2 values across transplant years were assessed using one-way ANOVA, while differences in overall community composition were evaluated using PERMANOVA with 999 permutations. Detailed results are provided in the Supplementary Materials (Supplementary Figures S2 and S3).

Additionally, the Mann–Whitney U test and Analysis of Similarities (ANOSIM) were used to compare differential abundance between patient groups.

Spearman’s rank correlation coefficient (ρ) was used to assess non-parametric associations between the relative abundance of microbial taxa and a range of clinical and prognostic markers. Given the exploratory nature of the microbiome analyses and the modest sample size, all statistical associations should be interpreted cautiously, and no causal inferences were drawn.

All analyses were observational in nature; therefore, the study was not designed to support causal inference. A two-tailed *p*-value of <0.05 was considered statistically significant.

### 2.7. Use of AI Tools

Generative AI tools were employed in the preparation of the manuscript text. Specifically, ChatGPT (GPT-5 mini, OpenAI; accessed 19 August 2025) was used to assist with English grammar, phrasing, and overall readability. All content produced with the assistance of this tool was carefully reviewed, verified for accuracy, and edited by the authors to ensure scientific rigor, reproducibility, and adherence to journal standards. The authors retain full intellectual responsibility for the final manuscript, including study design, data analysis, interpretation of results, and conclusions.

## 3. Results

### 3.1. Sample Inclusion and Patients’ Characteristics

Among the 169 patients who underwent LTx for PSC between 1987 and 2015, colon biopsy samples were available for 98 individuals, representing 58% of the cohort. In the remaining 71 cases (42%), formalin-fixed paraffin-embedded (FFPE) blocks could not be retrieved from the biobank due to insufficient material. Additionally, one patient’s sample was excluded during analysis because of a low read count in microbiome analysis. *FUT2* genotyping was successfully performed on samples from 87 patients (51% of the original cohort). These 87 patients were included in all subsequent analyses and characterizations (Supplementary Figure S1).

Of the 98 patients with available FFPE material, *FUT2* genotyping failed in 11 cases due to insufficient quantity or quality of host genomic DNA extracted from archival FFPE tissue, despite repeated amplification attempts.

*FUT2* genotype distribution and associated clinical outcomes are presented in Supplementary Table S1. Among the 87 genotyped patients, 28 (32%) were non-secretors (AA) and 59 (68%) were secretors (GA + GG), corresponding to 30 and 29 patients in the respective outcome groups. This genotype distribution is consistent with reported FUT2 allele frequencies in European populations.

Recurrent PSC occurred in 8 non-secretors (29%) compared with 6 secretors (10%), corresponding to a statistically significant association (*p* = 0.028). Consistently, rPSC was more frequent in *FUT2* non-secretors than in secretors (OR = 3.54, 95% CI 1.10–11.4), indicating a significant association between *FUT2* non-secretor status and rPSC. Post-transplant bacteremia was more frequent in non-secretors (17 patients, 61%) than in secretors (13 patients, 22%), also showing a significant association (*p* = 0.0019). Among patients who developed bacteremia, intestinal-type episodes were documented in 10 non-secretors (53%) and 9 secretors (47%), without a significant difference between groups (*p* = 0.71). In total, rPSC, bacteremia, and intestinal-type bacteremia occurred in 14, 30, and 19 patients, respectively, across the entire cohort (Supplementary Table S1).

Patient characteristics are detailed in Table 1. Age at PSC diagnosis was 34.7 ± 12 years, and the majority of patients (67%) were male. For all primary LTxs, liver cirrhosis served as the primary indication in 80% of cases, followed by recurrent cholangitis in the remaining 20 (Table 1). The mean duration of follow-up after LTx was 10.7 years, with a standard deviation of 6.0 years. After transplantation, rPSC was seen in 14 patients (16%) and was more frequent in males and in patients with IBD, particularly ulcerative colitis (UC). Due to the retrospective design and long study period, standardized IBD activity scores at the time of biopsy were not consistently available.

The diagnosis of rPSC occurred, on average, after 4.7 years (with a standard deviation of 3.5 years) post-LTx.

### 3.2. Assessment of Transplant–Era Effects

Given the 28-year inclusion period, we evaluated whether transplant year was associated with differences in baseline clinical characteristics or microbial community composition. No significant associations were observed between transplant year and major clinical variables, including sex, inflammatory bowel disease status, transplantation indication, or recurrent PSC (Supplementary Figure S2). Similarly, transplant year was not associated with variation in microbial community structure, as assessed by PCA (PC1: *p* = 0.538; PC2: *p* = 0.117) and Bray–Curtis-based PERMANOVA (*p* = 0.212) (Supplementary Figure S3). These findings suggest that transplant era was not a major source of variation within the study cohort.

### 3.3. FUT2 Genotype Is Associated with Colonic Microbiome Composition

**Figure 1:**
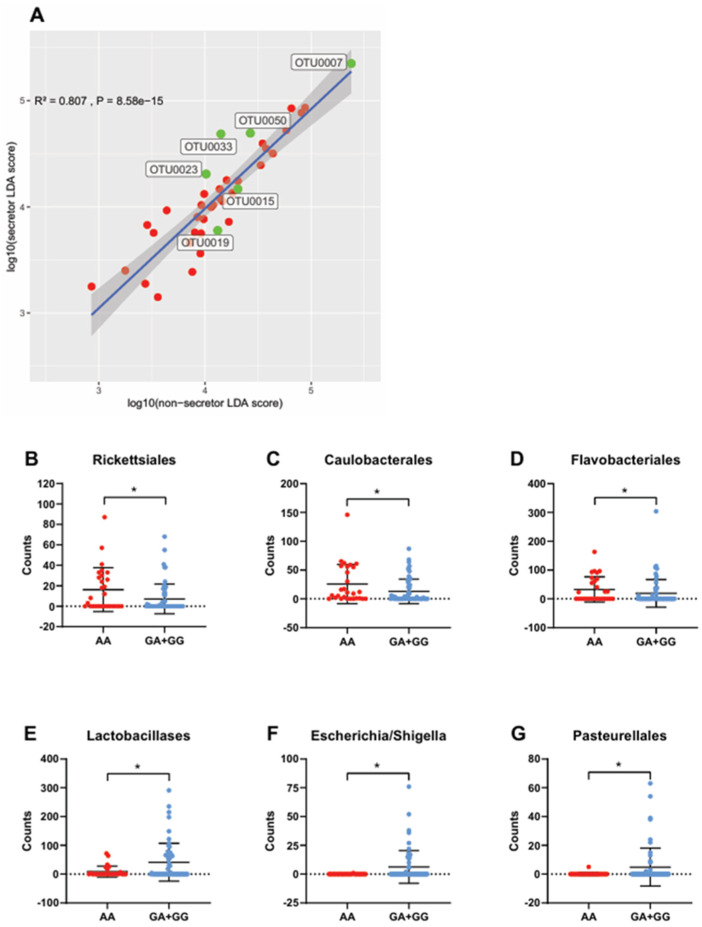
Association between secretor status-related LDA scores and gut microbial taxa stratified by genotype. (**A**) Relationship between log10-transformed LDA scores for non-secretor-associated and secretor-associated OTUs. Each point represents an OTU, with selected taxa highlighted and labeled. A strong positive correlation is observed between the two LDA score sets (linear regression: R^2^ = 0.807, *p* = 8.58 × 10^−15^), indicating concordant effect sizes across microbial features associated with secretor status. The fitted linear regression line is shown in blue with the 95% confidence interval shaded in grey. (**B**–**G**) Differential abundance of bacterial taxa at the order level stratified by genotype (AA vs. GA + GG). Red points represent individuals with genotype AA (*n* = 28), and blue points represent carriers of the GA + GG genotype (*n* = 59). Each dot represents an individual sample; horizontal bars indicate group means, and the dashed line denotes zero (baseline relative abundance or normalized count). Statistically significant differences between genotypes are indicated by asterisks * Statistical significance (*p* < 0.05) Mann–Whitney nonparametric U test).

We investigated microbiome composition in relation to *FUT2* status in PSC patients before LTx. Significant differences were observed in microbiome composition between *FUT2* non-secretors (AA genotype) and secretors (GA or GG genotypes) (Figure 1A). Given the use of FFPE-derived tissue and low microbial biomass, all microbiome analyses were interpreted as exploratory and hypothesis-generating. *FUT2* non-secretors displayed an increased abundance of certain taxa within *Proteobacteria*, particularly *Rickettsiales* (OTU0019, *p* = 0.04) and *Caulobacterales* (OTU0015, *p* = 0.01) (Figure 1B,C). Additionally, within the phylum *Bacteroidetes*, the *Flavobacteriales* order, including the genus *Tenacibaculum* (OTU0007, *p* = 0.04), was enriched in non-secretors (Figure 1D).

Conversely, several bacterial taxa were significantly reduced in *FUT2* non-secretors. Within *Firmicutes*, *Lactobacillales* exhibited a decreased relative abundance (OTU0050, *p* = 0.03) (Figure 1E), while within *Proteobacteria*, *Enterobacteriales*, particularly *Escherichia/Shigella*, showed a decrease in relative abundance (OTU0033, *p* = 0.006) (Figure 1F). Additionally, within *Proteobacteria*, the *Pasteurellales* order was less abundant in non-secretors compared to secretors (OTU0023, *p* = 0.03) (Figure 1G).

Overall, these exploratory findings may indicate differences in microbial composition associated with *FUT2* secretor status; however, the observed shifts in *Proteobacteria* and *Bacteroidetes* should be interpreted cautiously due to the limitations of FFPE-derived samples, low microbial biomass, and limited sequencing depth.

### 3.4. Pre-Transplant Colonic Microbiome Is Associated with Post-Transplant Bacteremia

Given the observed association between PSC recurrence and bacteremia [8], we next investigated whether bacteremia correlates with alterations in the gut microbiome. To explore whether microbial composition was associated with blood sampling itself, we compared the relative abundance of bacterial taxa between patients who had blood drawn and those who did not (Supplementary Figure S2). Several taxa were significantly more abundant in the blood-sampled group, including *Neisseriales*, *Micrococcaceae*, *Methylophilales*, *Flavobacteriales* and *Corynebacteriaceae*.

These differences likely reflect clinical indications for blood culture sampling rather than causal microbiome effects and were therefore not interpreted as biologically driven associations.

**Figure 2:**
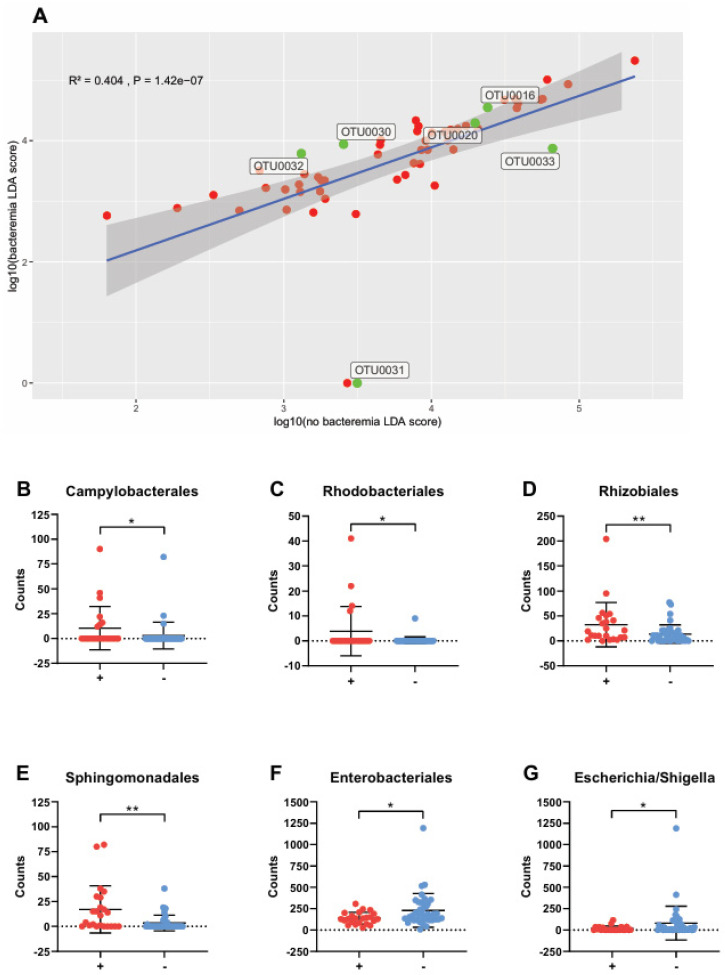
**Differentially abundant bacterial taxa associated with bacteremia status.** (**A**) Correlation between the log10-transformed linear discriminant analysis (LDA) scores of bacterial taxa in bacteremia-positive (green) (*n* = 30) and bacteremia-negative (red) samples (*n* = 57). Each point represents an operational taxonomic unit (OTU), with selected OTUs labeled. The blue line indicates the linear regression fit with the shaded area representing the 95% confidence interval. Correlation statistics are shown (R^2^ = 0.404, *p* = 1.42 × 10^−7^). (**B**–**G**) Relative counts of significantly different bacterial taxa between bacteremia-positive (*n* = 30, red) and bacteremia-negative (*n* = 57, blue) groups, including (**B**) *Campylobacterales*, (**C**) *Rhodobacteriales*, (**D**) *Rhizobiales*, (**E**) *Sphingomonadales*, (**F**) *Enterobacteriales*, and (**G**) *Escherichia/Shigella*. Each dot represents an individual sample; horizontal bars indicate mean ± SEM. Statistical significance was determined using Mann–Whitney nonparametric U test. Asterisks indicate significance levels (*p* < 0.05 *, *p* < 0.01 **).

Microbial profiling revealed distinct differences in the relative abundance of several bacterial taxa between patients with bacteremia and those without after LTx (Figure 2A). Patients who developed post-LTx bacteremia showed a significantly higher abundance of *Campylobacterales*, an order within the *Epsilonproteobacteria* class (OTU0030, *p* = 0.04) (Figure 2B), as well as *Rhodobacterales* from *Alphaproterobacteria*, which were also significantly enriched in culture-positive samples compared to culture-negative ones (OTU0031, *p* = 0.036) (Figure 2C). Additionally, within *Alphaproteobacteria*, there was notable enrichment in the *Rhizobiales* (OTU0016, *p* = 0.03) (Figure 2D) and *Sphingomonadales* orders (OTU0020, *p* = 0.0003) in this group (Figure 2E).

Conversely, significantly lower abundance of *Enterobacteriales* of the *Gammaproteobacteria* class was associated with bacteremia-positive recipients (OTU0032, *p* = 0.02) (Figure 2F). This was particularly prominent in *Escherichia/Shigella* (OTU0033, *p* = 0.01) (Figure 2G), mirroring some of the microbiome shifts observed in *FUT2* non-secretors.

The overlap between taxa associated with *FUT2* non-secretor status and bacteremia suggests shared dysbiotic features, although causality cannot be inferred from these observational data.

The observed bacteremia-associated microbial signatures should be interpreted cautiously, as potential confounding by *FUT2* genotype, biopsy location, IBD activity, transplant era, antibiotic exposure, and timing of bacteremia could not be fully adjusted for in this retrospective exploratory analysis.

Each bacteremia episode was further categorized according to its most likely source (intestinal or non-intestinal), with detailed classifications summarized in Supplementary Table S1. In addition, the observed microbiome differences between blood-sampled and non-sampled patients likely reflect differences in clinical indication for blood culture sampling rather than direct microbial causation (Supplementary Figure S2).

These findings are important because they highlight the potential for ascertainment bias in this retrospective cohort, as blood cultures were obtained based on clinical suspicion of infection rather than systematically across all patients. Therefore, microbiome differences between blood-sampled and non-sampled individuals may reflect underlying clinical status and sampling practices rather than biologically driven associations.

As with the blood-sampled versus non-sampled comparison, these findings should be interpreted cautiously because bacteremia-positive patients likely differed in clinical status and healthcare exposure, limiting causal interpretation of the observed microbiome associations.

### 3.5. FUT2 Genotype and Post-Transplant Bacteremia Are Associated with Survival After LTx

**Figure 3:**
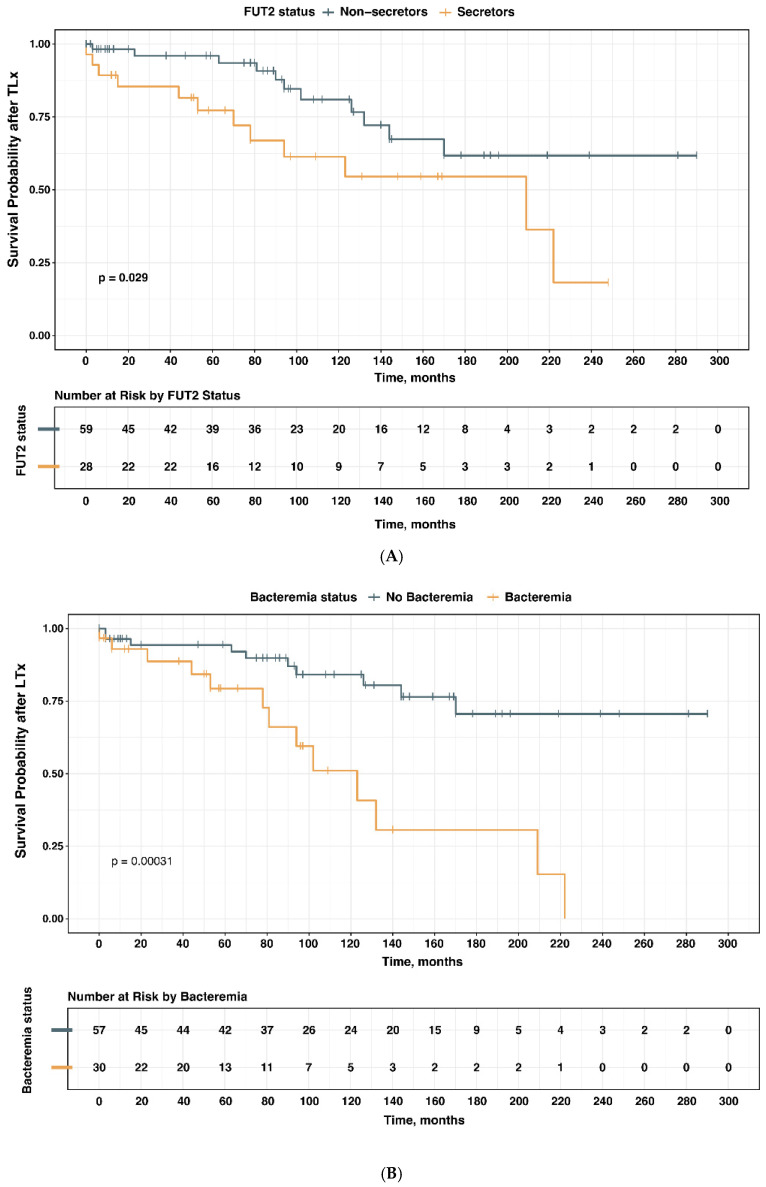
Relation between different FUT2 status, bacteremia and survival probability in PSC patients after LTx. (**A**) Survival probability in PSC patients in relation with different *FUT2* genotypes: PSC patients with non-secretor genotype (AA) have a significantly decreased survival compared with patients with a secretor status (GA + GG) (*p* = 0.029). (**B**) Survival probability in PSC patients in relation with bacteremia: PSC patients with positive bacteremia (*n* = 30) have a significantly decreased survival compared to in those without bacteremia (*n* = 24) or blood cultures performed (*n* = 33) (*p* = 0.0003).

Kaplan–Meier survival analysis revealed that recipient *FUT2* non-secretor status was significantly associated with decreased post-transplant overall survival (Log-Rank *p* = 0.029, Figure 3A).

Similarly, the presence of post-transplant bacteremia was linked to a significantly lower survival rate as compared to patients without or negative blood cultures (Log-Rank *p* < 0.001, Figure 3B). Kaplan–Meier analyses were used for descriptive visualization of survival differences between groups and should not be interpreted as fully adjusted survival estimates.

These findings demonstrate an association between *FUT2* genotype, post-transplant bacteremia, and long-term outcomes after LTx.

A proportion of patients were censored within the early post-transplant period, reflecting the extended inclusion window (1987–2015) and real-world clinical follow-up rather than disease-related mortality alone.

To further examine these associations, we conducted a binary logistic regression analysis (Table 2). Interestingly, the presence of rPSC in the univariable analysis was not significantly associated with decreased overall survival (OR = 2.471; 95% CI: 0.752–8.117; *p* = 0.163). In contrast, both post-transplant bacteremia (OR = 4.301; 95% CI: 1.575–11.743; *p* = 0.010) and *FUT2* non-secretor status (OR = 3.273; 95% CI: 1.210–8.849; *p* = 0.016) were significantly associated with decreased overall survival.

In the multivariable analysis, both post-transplant bacteremia and *FUT2* non-secretor status remained significant independent risk factors for reduced survival. Bacteremia was associated with decreased survival (OR = 3.226; 95% CI: 1.154–9.020; *p* = 0.030), as was *FUT2* non-secretor status (OR = 3.134; 95% CI: 1.145–8.575; *p* = 0.026).

### 3.6. Association of Post-Transplant Mortality with Pre-Transplant Colonic Microbiome

**Figure 4:**
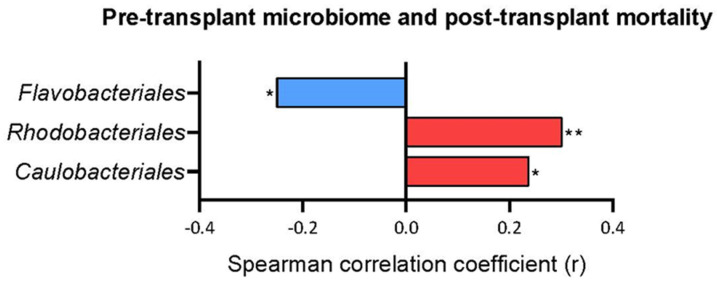
Spearman correlations between patient mortality and bacterial taxa abundance. Positive correlations were observed between mortality and the relative abundance of *Caulobacterales* (r = 0.237, *p* = 0.027) and *Rhodobacterales* (r = 0.301, *p* = 0.005), indicating a possible association with increased mortality risk. Conversely, mortality was negatively correlated with *Flavobacteriales* abundance (r = −0.250, *p* = 0.019), suggesting a potential protective role. Asterisks indicate significance levels (*p* < 0.05 *, *p* < 0.01 **).

Analysis of microbial community composition revealed significant correlations between specific bacterial taxa and patient mortality outcomes. Notably, the relative abundance of *Caulobacterales* (r = 0.237, *p* = 0.027) and *Rhodobacterales* (r = 0.301, *p* = 0.005) exhibited positive associations with mortality, indicating that higher levels of these taxa were observed in patients who subsequently experienced post-transplant death (Figure 4).

Conversely, *Flavobacteriales* abundance was negatively correlated with mortality (r = −0.250, *p* = 0.019), suggesting that higher levels of this group may be associated with improved survival or reflect a more balanced microbial community.

These correlations should be interpreted as associative rather than predictive, given the observational and exploratory nature of the study.

The positive correlations with *Caulobacterales* and *Rhodobacterales* may reflect broader dysbiotic shifts linked to systemic vulnerability or impaired host defenses, rather than direct pathogenicity of these taxa. Conversely, the inverse relationship with *Flavobacteriales* may indicate a role in maintaining microbial homeostasis or modulating host immune responses beneficially.

Overall, these findings suggest that microbial community signatures are associated with clinical outcomes and may warrant further investigation in future prognostic and mechanistic studies. However, causality cannot be inferred, and no predictive or clinically validated biomarker performance can be concluded from the present exploratory analysis. Although statistically significant, the observed correlation coefficients were modest and should therefore be interpreted cautiously.

### 3.7. Associations Between Amsterdam Oxford Model, Mayo Risk Score, and Pre-Transplant Colonic Microbiome

**Figure 5:**
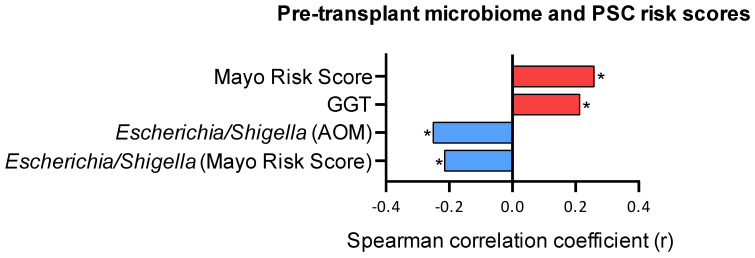
Spearman correlation analysis between the Amsterdam Oxford Model (AOM), Mayo Risk Score, and pre-transplant microbiome. A positive correlation was observed between the AOM score and the Mayo Risk Score (r = 0.259, *p* = 0.015, *n* = 87), as well as between the AOM score and GGT levels (r = 0.214, *p* = 0.047, *n* = 87). A negative correlation was identified between the AOM score and the relative abundance of *Escherichia/Shigella* (r = −0.251, *p* = 0.019, *n* = 87). Similarly, the Mayo Risk Score was negatively correlated with the relative abundance of *Escherichia/Shigella* (r = −0.215, *p* = 0.045, *n* = 87). Asterisks indicate significance levels (*p* < 0.05 *).

The Amsterdam Oxford Model (AOM), a prognostic scoring system for primary sclerosing cholangitis, demonstrated a modest but statistically significant positive correlation with the Mayo Risk Score (r = 0.259, *p* = 0.015) and gamma-glutamyl transferase (GGT; r = 0.214, *p* = 0.047), indicating that higher AOM values are associated with increased cholestatic liver injury and worse clinical prognosis. GGT is an enzyme linked to bile duct injury and cholestasis, and its elevation reflects ongoing hepatobiliary damage (Figure 5).

Both AOM and Mayo Risk Score were negatively correlated with the relative abundance of *Enterobacteriales_Escherichia/Shigella* taxa (r = −0.251, *p* = 0.019 and r = −0.215, *p* = 0.045, respectively), suggesting that a reduction in these specific gut bacteria may be related to more severe disease states.

Given the observational nature of this study, these correlations should not be interpreted as causal; the findings may reflect broader dysbiotic states linked to host disease severity rather than direct microbial effects.

Overall, these results highlight a potential interplay between microbial dysbiosis and established clinical risk scores, emphasizing that gut microbiome alterations may serve as associative markers of disease progression or prognosis in PSC, rather than direct determinants of clinical outcome.

## 4. Discussion

This study highlights the intricate interplay among the *FUT2* genotype, pre-transplant mucosal microbiome alterations, bacteremia, and patient mortality following LTx. Our findings reveal significant associations between genetic and microbial factors, disease progression, and mortality risk, providing exploratory insights relevant for future mechanistic and therapeutic studies.

Importantly, all observed associations should be interpreted within the context of an observational, retrospective study design, and do not imply causality. An altered microbiome associated with *FUT2* non-secretor status may be linked to impaired barrier function and increased frequency of bacteremia, which could contribute to adverse outcomes after LTx.

Dysbiosis in IBD patients is characterized by a reduction in *Firmicutes* and *Bacteroidetes*, along with an increased abundance of *Proteobacteria*, which includes many opportunistic pathogens [24]. This microbial imbalance is associated with increased intestinal permeability, chronic inflammation, and bacterial translocation, all of which are critical factors in the pathogenesis of PSC [20,27,28,29]. IBD, particularly UC, is highly prevalent among PSC patients and further confirms that IBD is a major contributing factor to PSC [30,31], supporting previous studies that demonstrate the interconnected nature of gut–liver axis dysfunction in these diseases [32,33]. Although detailed IBD activity scores and medication use were not uniformly available due to the long inclusion period, biopsies were obtained during clinically stable disease, reducing but not eliminating potential confounding by active inflammation.

The *FUT2* gene plays a central role in the regulation of the microbiome composition through its influence on the production of fucosylated glycans, which are secreted into mucosal surfaces of the gut [12,19]. Both IBD and PSC are associated with genetic variants in the *FUT2* gene, and our previous study demonstrated a link between PSC recurrence and the non-secretor (AA) genotype [8]. FUT2-dependent fucosylation contributes to the generation of mucosal glycans that serve both as attachment substrates and metabolic nutrients for commensal microorganisms [34,35]. Loss of FUT2 activity in non-secretors may therefore alter microbial niche selection, reduce colonization by glycan-utilizing commensals, and favor expansion of opportunistic or inflammation-associated taxa [36,37]. In addition, altered mucosal glycosylation may impair epithelial barrier integrity and modify host–microbe signaling pathways involved in innate immune sensing, antimicrobial defense, and mucosal immune tolerance [38,39]. Such alterations may contribute to increased bacterial translocation, chronic intestinal inflammation, and activation of the gut–liver axis in PSC.

The present study extends these observations by linking *FUT2* genotype to mucosal microbiome composition derived from archival tissue, representing a novel methodological approach despite inherent technical constraints.

Notably, our analysis revealed significant alterations in gut microbiota composition linked to *FUT2* non-secretor status. Individuals with the non-secretor genotype exhibited a distinct microbial profile marked by an increased abundance of *Proteobacteria*, particularly *Alphaproteobacteria*, including *Rickettsiales* and *Caulobacterales* taxa, previously associated with impaired epithelial barrier function and elevated inflammatory responses [40,41]. Elevated levels of *Flavobacteria* and *Flavobacteriales*, including *Tenacibaculum*, were also observed in non-secretors, indicating possible shifts toward biofilm-forming and tissue-colonizing microbial communities [42,43,44]. Such bacteria have previously been associated with local inflammation and immune modulation, further supporting a potential association between dysbiosis and disease pathogenesis [45]. Non-secretors also showed a decrease in *Lactobacillales* and *Enterobacteriales*, including *Escherichia/Shigella*. Although members of *Enterobacteriales* may include opportunistic pathogens, depletion of specific strains or commensal-associated members of this taxonomic group may also reflect broader disturbances in microbial ecosystem stability and mucosal immune homeostasis [39,45,46]. Given the taxonomic resolution limitations of 16S rRNA sequencing, these findings should not be interpreted as evidence for loss of uniformly beneficial organisms, but rather as indicators of altered microbial community structure.

Given the use of FFPE-derived samples, low microbial biomass, and limited sequencing depth, these microbiome findings should be considered exploratory and hypothesis-generating rather than definitive.

Our findings suggest that *FUT2* non-secretor status is associated with a higher frequency of positive bacteremia. We also observed distinct microbiome shifts in bacteremia-positive patients, notably an increase in *Alphaproteobacteria* (*Rhizobiales*, *Sphingomonadales* and *Rhodobiales*) as well as an enrichment in *Epsilonproteobacteria* (*Campylobacterales*, including *Helicobacteraceae*).

These changes suggest a shift toward opportunistic and potentially pathogenic taxa, which may exacerbate systemic inflammation and contribute to disease progression [47,48,49,50]. Their enrichment may be associated with bacterial translocation, systemic inflammation, and bile duct injury—key mechanisms in PSC pathogenesis [50,51].

However, these taxa may also represent broader signatures of altered mucosal ecology, immune dysregulation, antibiotic exposure, advanced cholestatic disease, or low-biomass background contamination rather than direct pathogenic drivers. In particular, taxa within *Alphaproteobacteria*, including *Sphingomonadales* and related environmental organisms, have previously been reported in low-biomass microbiome studies and may reflect both technical and biological influences [52,53]. Consequently, the observed enrichment patterns should be interpreted cautiously and viewed primarily as associative microbial signatures rather than definitive pathogenic organisms.

One particularly intriguing finding was the significant reduction in *Enterobacteriales*, including *Escherichia/Shigella*, in bacteremia-positive patients. This shift mirrors the changes observed in *FUT2* non-secretors and in patients with recurrence of PSC, suggesting this to be one of the most strongly associated bacterial traits [24]. The consistent depletion of this taxon across multiple adverse outcomes suggests it may represent a broader dysbiotic signature rather than a single disease-specific effect.

Kaplan–Meier survival analyses demonstrated descriptive differences in post-LTx survival according to *FUT2* genotype and bacteremia status. These associations remained significant in multivariable regression analyses, although the observational study design and limited covariate adjustment preclude definitive causal or fully adjusted survival inference. Interestingly, rPSC, although often implicated in poorer outcomes, did not emerge as a statistically significant predictor of mortality in this cohort, most likely due to the limited sample size [54]. Furthermore, the observed pattern of censoring is likely influenced by the long study period (1987–2015) and routine clinical follow-up, rather than reflecting transplant-related mortality alone.

The data presented here highlight the clinical importance of post-transplant infections, with bacteremia conferring more than a threefold increased risk of mortality. In parallel, the *FUT2* non-secretor genotype, known to influence mucosal glycosylation and microbiome composition [16,19], was also significantly associated with decreased survival. These results suggest that both infectious complications and host genetic susceptibility are associated with poor long-term outcomes after transplantation. Nonetheless, residual confounding by unmeasured variables such as donor characteristics, evolving immunosuppressive regimens, and antibiotic exposure during follow-up cannot be fully excluded.

Microbial profiling further supported the link between host and microbial factors in shaping prognosis. Notably, *Caulobacterales* and *Rhodobacterales* were positively correlated with mortality, suggesting that enrichment of these taxa may reflect a dysbiotic shift associated with systemic inflammation, immune dysfunction, or microbial translocation. Importantly, *Caulobacterales* was also more abundant in *FUT2* non-secretors, while *Rhodobacterales* was enriched in bacteremia-positive patients providing a potential mechanistic link between host genotype, microbial ecology, and infection risk. These associations suggest that microbial patterns linked to host factors may reflect broader biological processes associated with disease progression and transplant outcomes. However, given the screening-level resolution of 16S rRNA sequencing and multiple taxonomic comparisons, these associations should be interpreted cautiously.

Additionally, correlations between microbial signatures and established prognostic models such as the Amsterdam–Oxford Model (AOM) and Mayo Risk Score suggest that dysbiosis may be integrated into clinical risk assessment frameworks.

Both AOM and Mayo Risk Score were inversely correlated with *Enterobacteriales_Escherichia/Shigella* abundance, a taxonomic group often depleted in *FUT2* non-secretors and bacteremia patients. This further supports a possible association between depletion of specific microbial taxa, altered intestinal barrier function, and immune dysregulation contributing to worse clinical trajectories. Whether such microbial alterations precede disease progression or arise as a consequence of advancing disease remains to be determined.

In addition to epithelial barrier dysfunction and innate immune activation, adaptive immune mechanisms may also contribute to the observed associations between *FUT2* genotype, dysbiosis, and post-transplant outcomes. PSC is increasingly recognized as an immune-mediated cholangiopathy characterized by dysregulated T helper cell responses, including enhanced Th1- and Th17-associated inflammation alongside impaired regulatory T-cell (Treg) activity [55,56]. Altered microbial composition and bacterial-derived extracellular signals may influence mucosal antigen presentation, cytokine production, and epithelial immune activation, thereby promoting chronic hepatobiliary inflammation and fibrosis. FUT2-associated alterations in microbial ecology may therefore contribute not only to increased bacteremia susceptibility, but also to maladaptive immune signaling pathways involved in PSC progression and post-transplant inflammatory complications [55,57].

Our data provide rationale for future investigation of microbiome-associated interventions and strategies aimed at restoring a more balanced microbial ecosystem, particularly by promoting beneficial bacteria and suppressing inflammatory or opportunistic pathogens [58,59]. The association of *FUT2* non-secretor status with dysbiosis, bacteremia, and post-transplant mortality also offers valuable insights for risk stratification and personalized management of PSC patients undergoing LTx, supporting further investigation into microbiome-associated biomarkers potentially linked to bacteremia and mortality as well as strategies for microbiome modulation, barrier enhancement, and enhanced monitoring of at risk individuals. These insights could lead to targeted interventions, including the use of probiotics, prebiotics, or other microbiome-based therapies, to optimize outcomes for PSC patients.

A key limitation is that blood cultures were not obtained systematically but only in patients with clinical suspicion of infection. Therefore, absence of recorded bacteremia reflects absence of documented microbiological testing rather than confirmed absence of infection. This likely introduces ascertainment bias and may have led to underestimation of bacteremia incidence and biased associations with microbiome features and outcomes.

An additional limitation is that the timing and frequency of blood culture sampling were determined by treating physicians based on clinical indication rather than being standardized across the cohort. This introduces variability in bacteremia ascertainment and may have resulted in heterogeneity in detection rates, as patients with more severe clinical presentations were more likely to undergo microbiological testing.

An additional important limitation is the potential for residual confounding due to clinical and treatment-related variables that were not fully available or consistently recorded in this retrospective cohort. Survival and post-transplant bacteremia are influenced by multiple factors, including transplant era, donor characteristics, biliary complications, perioperative and long-term antibiotic exposure, immunosuppressive regimens, patient age, and IBD activity. Given the long study period (1987–2015) and evolving clinical practice over time, not all of these variables could be systematically included in adjusted analyses. Therefore, despite multivariable modeling of available covariates, residual confounding cannot be excluded, and the observed associations should be interpreted in this context.

Although no significant transplant-era effects were identified, residual confounding due to changes in clinical practice over the study period cannot be excluded.

Standardized IBD activity indices, fecal calprotectin levels, and complete longitudinal CRP or histological inflammation scores were not systematically available for most patients due to the retrospective design and long inclusion period; therefore, residual confounding by subclinical mucosal inflammation cannot be excluded.

Additionally, several taxa identified in this study, including *Sphingomonadales*, have been reported as potential reagent- or environment-associated contaminants in low-biomass microbiome analyses. As systematic negative-control sequencing data were not available, these findings should be interpreted with caution.

The relationship between intestinal dysbiosis, bacteremia, and liver disease progression in PSC is likely complex and bidirectional. In addition to impaired intestinal barrier integrity, dysfunction of the hepatic reticuloendothelial system, including Kupffer cell-mediated bacterial clearance, may contribute to bacteremia and systemic endotoxinemia in advanced cholestatic liver disease. Furthermore, microbiome alterations may both contribute to and arise from progressive liver pathology and systemic inflammation. Therefore, the causal directionality of the observed associations cannot be determined from this retrospective observational study.

Future multicenter, prospective studies using fresh-frozen tissue, shotgun metagenomics, and integrated host–microbiome profiling will be important to validate these associations and clarify their potential clinical relevance.

## Figures and Tables

**Table 1:** Characteristics of patients included in FUT2 analysis.

Characteristic	Participants (*n* = 87)	FUT2		Bacteremia	
		Non-Secretors (AA) (*n* = 28)	Secretors (GA + GG) (*n* = 30 + 29)	Yes (*n* = 30)	No (*n* = 57)
**Age at Dx (yrs) mean (SD)**	34.7 ± 12.66	32.5 ± 15.1	35.8 ± 11.3	33.7 ± 15.0	35.3 ± 11.3
**Age at LTx (yrs) mean (SD)**	44.21 ± 11.91	41.9 ± 13.8	45.3 ± 10.8	43.8 ± 14.6	44.4 ± 10.3
**CIT (min)** **median (IQR)**	434.0 (341.5–542.5)	469.5 (365.3–542.3)	424.0 (333.0–544.0)	460.0 (337.0–521.5)	425.0 (344.0–543.0)
**WIT (min)** **median (IQR)**	28.0 (25.0–38.5)	31.0 (25.0–36.0)	28.0 (23.5–39.0)	27.0 (24.0–34.0)	31.0 (25.0–49.0)
**MELD score** **median (IQR)**	15.0 (12.0–19.0)	15.0 (12.0–19.0)	16.0 (11.5–19.0)	13.5 (10.3–17.8)	16.0 (12.0–19.0)
**Gender Male** ***n* (%)**	58 (67)	22 (78)	36 (61)	20 (67)	38 (67)
**Indication for LTx**					
**Cirrhosis (%)**	69 (79)	18 (64)	51 (86)	22 (73)	48 (84)
**Recurrent cholangitis (%)**	18 (21)	10 (36)	8 (14)	8 (27)	9 (16)
**Biopsy location**					
**Right hemicolon (%)**	41(53)	14 (50)	27 (46)	21 (70)	20 (35)
**Left hemicolon** **(%)**	34 (35)	10 (36)	24 (41)	8 (27)	26 (46)
**Unspecified colon (%)**	12 (12)	4 (14)	8 (13)	1 (3)	11 (19)
**IBD (%)**	72 (83)	21 (75)	51 (86)	21 (70)	41 (72)
**IBD type**					
**UC (%)**	51 (59)	18 (64)	33 (56)	19 (63)	32 (56)
**CD (%)**	11 (12)	3 (11)	8 (14)	2 (7)	9 (16)
**Graft type**					
**LD (%)**	3 (3)	0 (0)	3 (5)	1 (3)	2 (4)
**DBD (%)**	76 (89)	24 (86)	52 (88)	25 (83)	51 (89)
**DCD (%)**	8 (8)	4 (14)	4 (7)	4 (4)	4 (7)
**PSC type**					
**Large (%)**	84 (97)	27 (96)	57 (97)	29 (97)	55 (96)
**Small (%)**	3 (3)	1 (4)	2 (3)	1 (3)	2 (4)
**Biliary anastomosis type**					
**DD (%)**	16 (18)	4 (14)	12 (20)	7 (23)	9 (16)
**Roux en J (%)**	71 (82)	24 (86)	47 (80)	23 (77)	48 (84)

Abbreviations: PSC, primary sclerosing cholangitis; Dx, diagnosis; LTx, liver transplantation; CIT, cold ischemia time; WIT, warm ischemia time; MELD, model for end-stage liver disease; IBD, inflammatory bowel disease; UC, ulcerative colitis; SD, standard deviation; DBD, donation after brain death; DCD, donation after circulatory death; DD, duct to duct biliary anastomosis. Data are presented as mean ± standard deviation (SD), median (interquartile range, IQR), or number (percentage), as appropriate.

**Table 2:** Binary regression analysis for potential risk factors for overall post-transplant survival.

Variables	Univariable Analysis			Multivariable Analysis		
	OR	95%CI	*p*-Value	OR	95%CI	*p*-Value
**rPSC**	2.471	0.752–8.117	0.163	-	-	-
**IBD**	1.211	0.383–3.830	0.745	-	-	-
**FUT2 non-secretor vs. secretor**	3.273	1.210–8.849	**0.016**	3.134	1.145–8.575	**0.026**
**Post-transplant bacteremia: yes vs** **.** **no**	3.594	1.331–9.708	**0.010**	3.226	1.154–9.020	**0.030**

Binary regression analysis of potential risk factors for the posttransplant death including the combined factors of bacteremia and FUT2 status. Factors from univariable analysis with a *p*-value < 0.05 were included for multivariable analysis. CI, confidence interval, OR, odds ratio, FUT2, fucosyltransferase-2, rPSC, recurrence of primary sclerosing cholangitis, IBD, Inflammatory Bowel Disease. *p*-values < 0.05 considered statistically significant and are presented in bold.

## Data Availability

The data, analytic methods and study materials will be made available upon reason-able request.

## Supplementary Materials

The following supporting information can be downloaded at: https://www.mdpi.com/article/10.3390/jcm15124755/s1. Figure S1: Characterizations and subsequent analysis of patients included to the study (*n* = 87); Figure S2: Association of clinical variables with principal component structure of the cohort; Figure S3: PC1 and PC2 across transplant years; Table S1: Association of FUT2 genotype with the incidence of rPSC, bacteremia, type of bacteremia and mortality.

## Abbreviations

AOM	Amsterdam–Oxford Model
ANOSIM	Analysis of Similarities
CD	Crohn’s Disease
CIT	Cold Ischemia Time
CI	Confidence Interval
DBD	Donation After Brain Death
DCD	Donation After Circulatory Death
DD	Duct-to-Duct (biliary anastomosis)
FFPE	Formalin-Fixed Paraffin-Embedded
FUT2	Fucosyltransferase-2
GGT	Gamma-Glutamyl Transferase
IBD	Inflammatory Bowel Disease
LDA	Linear Discriminant Analysis
LD	Living Donor
LTx	Liver Transplantation
Log-Rank	Log-Rank test (statistical survival analysis)
Mayo Risk Score	Clinical prognostic score for PSC
MELD	Model for End-Stage Liver Disease
OUT	Operational Taxonomic Unit
OR	Odds Ratio
PSC	Primary Sclerosing Cholangitis
rPSC	Recurrence of Primary Sclerosing Cholangitis
SD	Standard Deviation
SNP	Single Nucleotide Polymorphism
UC	Ulcerative Colitis
WIT	Warm Ischemia Time
