# Supplementary material for: Association of FUT2 rs601338 Genotype with Colonic Mucosal Microbiome Composition, Post-Transplant Bacteremia, and All-Cause Mortality After Liver Transplantation for Primary Sclerosing Cholangitis: A Retrospective Cohort Study
Source: J Clin Med. 2026 Jun 18;15(12):4755. doi: 10.3390/jcm15124755 (PMC13301820; doi:10.3390/jcm15124755)
Supplement: Supplementary file 1 [file jcm-15-04755-s001.zip › jcm-4305255-supplementary.pdf]

Supplementary Figure S1. Characterizations and subsequent analysis of patients included to the study (n=87)

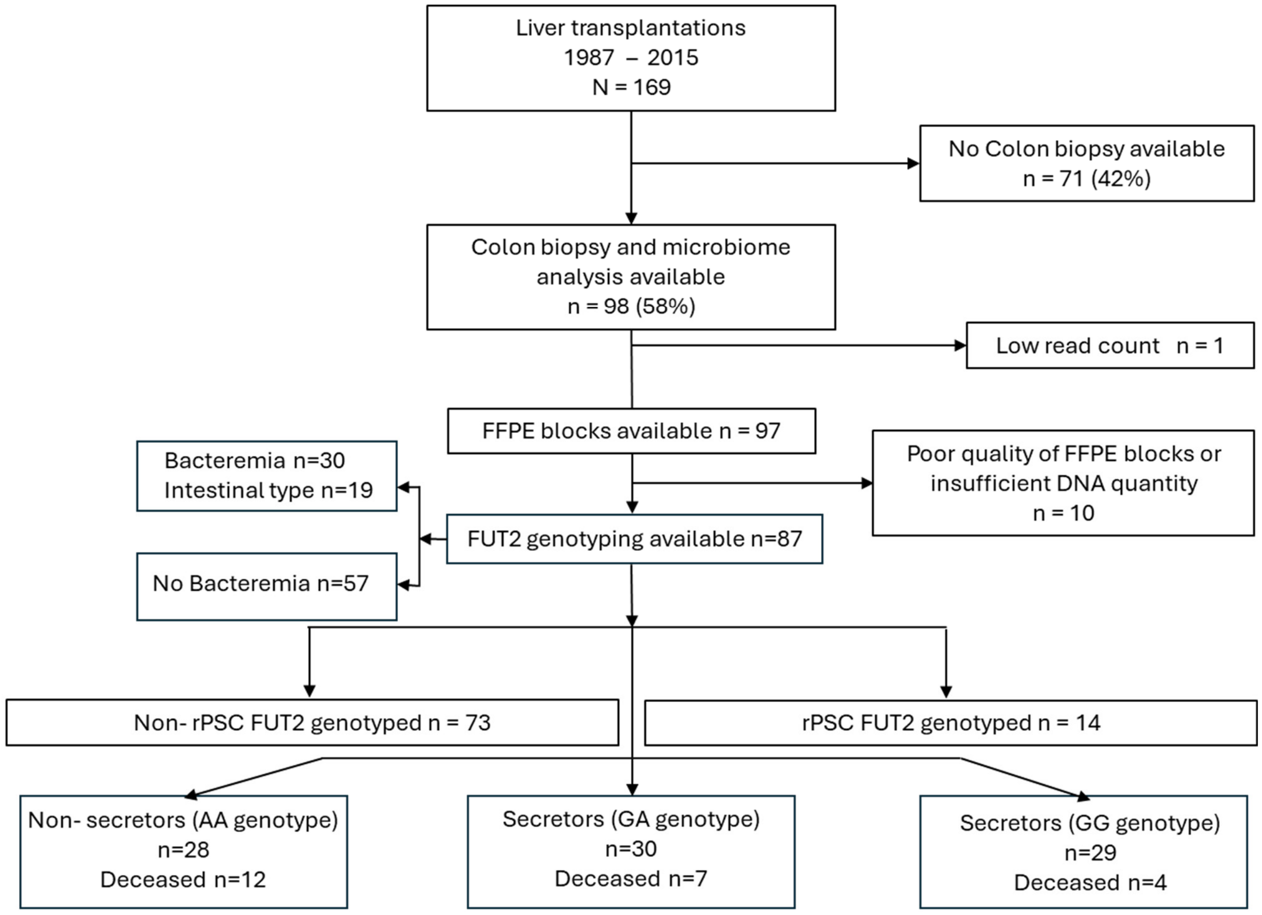

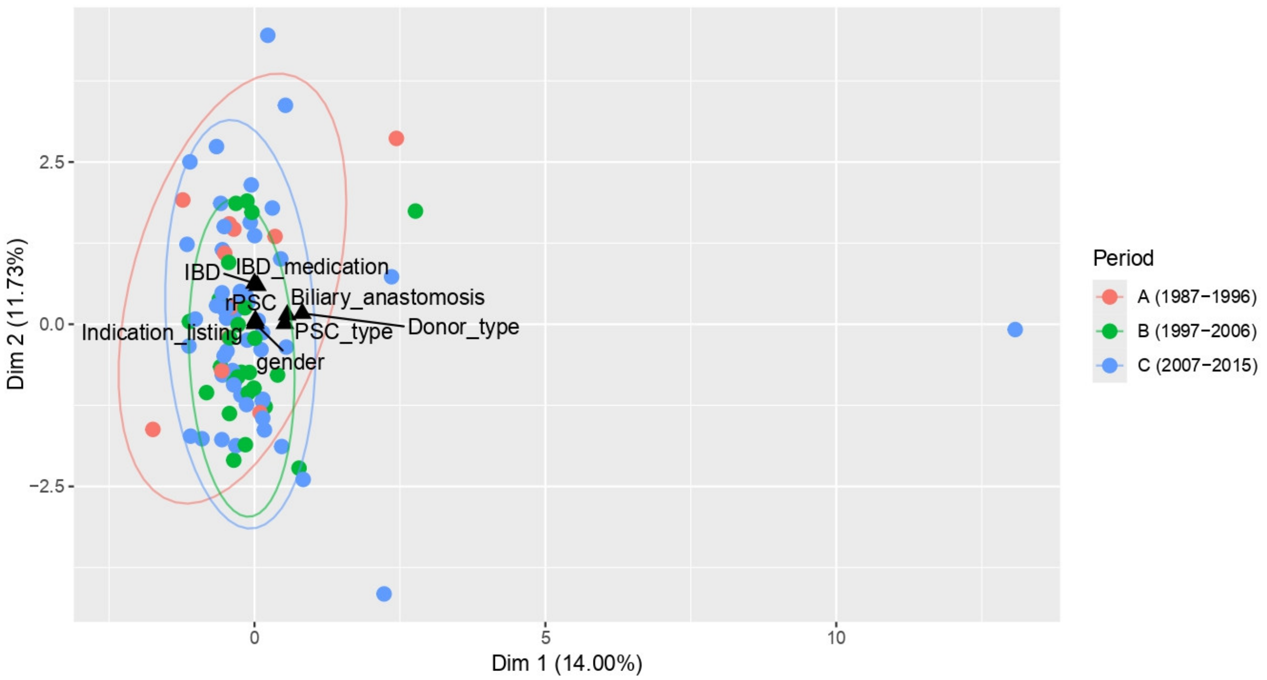

Supplementary Figure S2. Association of clinical variables with principal component structure of the cohort.

Principal component analysis (PCA) of baseline clinical characteristics in patients undergoing liver transplantation for primary sclerosing cholangitis (PSC). Variables shown include inflammatory bowel disease (IBD) status, IBD medication use, recurrent PSC (rPSC), biliary anastomosis type, indication for transplantation, PSC subtype, sex, and donor type. Samples are distributed along the first principal component (Dim 1, 14.0% explained variance). No clear clustering of clinical variables was observed along the principal component axis, supporting the absence of strong associations between transplant era–related cohort characteristics and the underlying structure of the dataset.

A

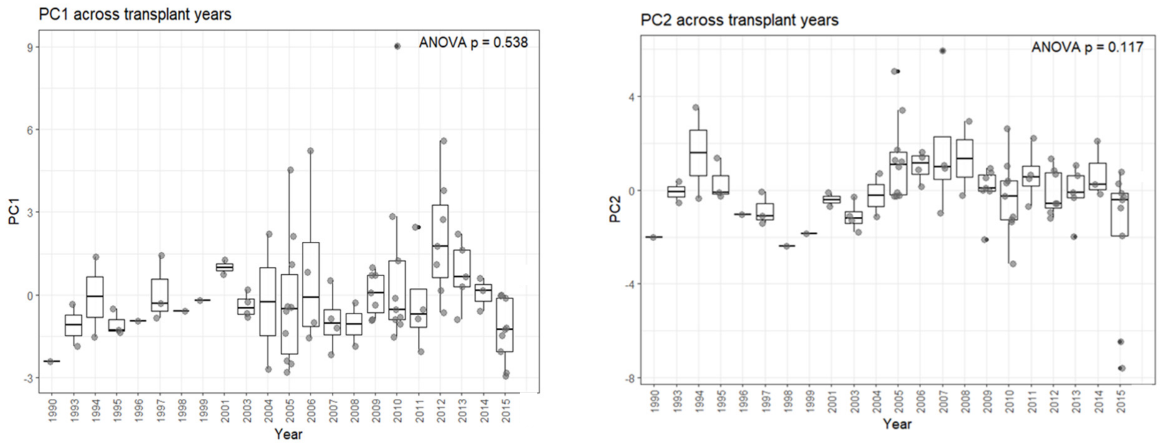

B

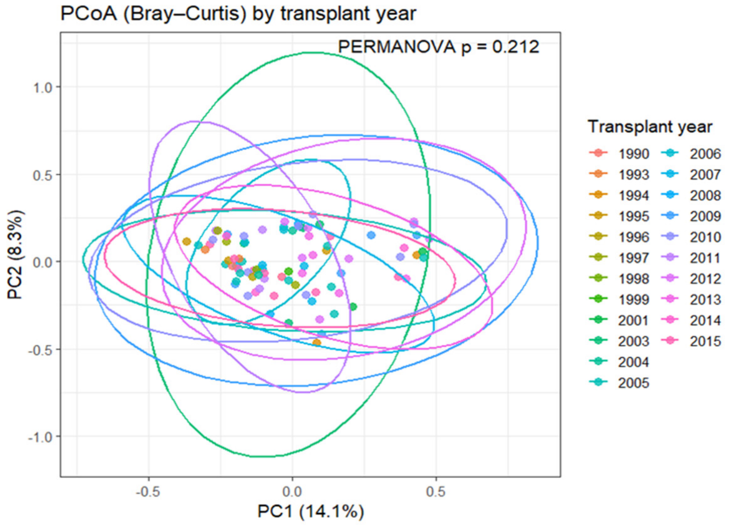

Supplementary Figure S3. PC1 and PC2 across transplant years.

A. Boxplots of PC1 and PC2 values derived from principal component analysis (PCA) of microbiome absolute count data, stratified by transplant year. Gray points represent individual samples. The ANOVA p-values indicate whether PC1 and PC2 differ significantly across transplant years.

B. PCoA of Bray–Curtis distances by transplant year.

Principal coordinates analysis (PCoA) based on Bray–Curtis dissimilarity of microbial community composition. Points are colored by transplant year, and 95% normal ellipses

illustrate group dispersion where sample size permits. The PERMANOVA p-value indicates whether overall community composition differs across transplant years.

Supplementary Table S1. Association of FUT2 genotype with the incidence of rPSC, bacteremia, type of bacteremia and mortality

FUT2 genotype	General cohort n, (%)	rPSC n, (%)	Bacteremia n, (%)	Intestinal-type bacteremia n, (%)	Deceased n, (%)
AA (Non-secretors)	28 (32%)	8 (29%)	17 (61%)	10 (36%)	12 (46%)
GA+GG (Secretors)	59 (68%)	6 (10%)	13 (22%)	9 (15%)	11 (19%)
Total N, (%)	87 (100%)	14 100%)	30 (100%)	19 (100%)	23 (100%)
P value (Chi-square test)		P=0.035*	P=0.0006*	P=0.038*	P=0.02*
